# Microbial Decontamination of *Cuminum cyminum* Seeds Using “Intensification of Vaporization by Decompression to the Vacuum”: Effect on Color Parameters and Essential Oil Profile

**DOI:** 10.3390/foods13142264

**Published:** 2024-07-18

**Authors:** Hana Tannir, Espérance Debs, Georges Mansour, Susanne Neugart, Rima El Hage, Mahmoud I. Khalil, Nada El Darra, Nicolas Louka

**Affiliations:** 1 Department of Biological Sciences, Faculty of Science, Beirut Arab University, Beirut P.O. Box 11-5020, Lebanon; hana.tannir@bau.edu.lb (H.T.); m.khalil@bau.edu.lb (M.I.K.); 2 Department of Biology, Faculty of Arts and Sciences, University of Balamand, Tripoli P.O. Box 100, Lebanon; esperance.debs@balamand.edu.lb; 3 Food Department, Lebanese Agricultural Research Institute, Fanar P.O. Box 2611, Lebanon; georges.mansour@gmail.com; 4 Division of Quality and Sensory of Plant Products, Department of Crop Sciences, Georg-August-Universität Göttingen, 37073 Göttingen, Germany; susanne.neugart@uni-goettingen.de; 5 Food Microbiology Laboratory, Lebanese Agricultural Research Institute (LARI), Fanar Station, Jdeideh El Metn P.O. Box 90-1965, Lebanon; relhage@lari.gov.lb; 6 Molecular Biology Unit, Department of Zoology, Faculty of Science, Alexandria University, Alexandria 21568, Egypt; 7 Department of Nutrition and Dietetics, Faculty of Health Sciences, Beirut Arab University, Beirut P.O. Box 11-5020, Lebanon; 8 Unité de Recherche Technologies et Valorisation Agro-Alimentaire, Centre d’Analyses et de Recherche, Faculté des Sciences, Saint-Joseph University of Beirut, Riad El Solh, P.O. Box 17-5208, Beirut 1104-2020, Lebanon

**Keywords:** *Cuminum cyminum*, IVDV, decontamination, response surface methodology, essential oils, GC-MS

## Abstract

Cumin seeds are frequently utilized in herbal infusions and as flavoring agents in home cuisine. Nevertheless, studies have demonstrated that spices are frequently contaminated with pathogenic bacteria, including bacterial spores. The aim of this study was to assess the effectiveness of a new decontamination method called “Intensification of Vaporization by Decompression to the Vacuum” (IVDV) on intentionally contaminated *Cuminum cyminum* seeds. The study also examined the impact of this treatment on the color and oil profile of the treated samples. The untreated samples were inoculated with *Escherichia coli* (ATCC 25922) and *Salmonella* Typhimurium (ATCC 14028) and then subjected to IVDV treatment. Response surface methodology was employed to obtain safe, high-quality cumin seeds presenting a balance between microbial load, color, and oil profile. The optimal IVDV conditions were achieved at a pressure of 3.5 bar and a time of 133.45 s, resulting in typical 4 log reductions observed with 99.99% of *Escherichia coli* and *Salmonella* Typhimurium inactivation. The treated spices presented a mild color modification compared to the untreated ones, manifested by a darker shade (decreased L* value), reduced greenness (increased a* value), and heightened yellowness (increased b* value). The GC-MS analysis detected the existence of seven compounds in the treated cumin, with cuminaldehyde being the primary compound (83.79%). Furthermore, the use of IVDV treatment resulted in an increase in the total content of essential oils in some samples, whereby six monoterpenes were identified in the untreated sample compared to seven monoterpenes in IVDV-treated samples. This innovative technology demonstrated high efficacy in decontaminating *C. cyminum* seeds, improving the extractability of the essential oils while only slightly affecting the color.

## 1. Introduction

Spices and herbs are vital ingredients in the human diet, serving as both flavor enhancers and therapeutic agents. In addition to their distinct flavor profiles, herbs and spices are commonly used as natural preservatives in food due to their significant antioxidant and antibacterial properties [1,2,3,4,5,6,7,8]. A study conducted by Sun et al. [9] showed that cuminic acid extracted from *C. cyminum* had high antifungal activity against *Fusarium oxysporum* f. sp. *niveum*. Furthermore, multiple researches have emphasized the anti-inflammatory and anticancer characteristics of herbs and spices, which contribute to their various therapeutic effects [10,11,12,13,14,15,16,17]. For instance, Goodarzi et al. [15] showed that luteolin-7-O-glucoside, a major flavonoid found in *C. cyminum*, demonstrated potent anticancer activities against breast cancer cell lines (MCF-7) and can be introduced as a candidate for chemopreventive and chemotherapeutic drugs. On the other hand, and despite the fact that spices typically have a low moisture content, which limits bacterial development, the conventional methods used to produce spices can result in contamination during harvesting, food handling, and transportation, contributing to foodborne infections and outbreaks [18,19,20,21]. This contamination can result in border rejection, which in turn can create trade hurdles for the export of spices and herbs. According to the EU Rapid Alert System for Food and Feed (RASFF) on herbs and spices, the top 10 notified herbs and spices during the last 23 years were chili, black pepper, curry, paprika, nutmeg, spice mix, basil, mint, ginger, and cumin, while the top 10 hazards were *Salmonella*, aflatoxin, Sudan 1, Sudan 4, ethylene oxide (EtO), ochratoxin A, chlorpyrifos, *Escherichia coli*, pyrrolizidine alkaloids, and food coloring E160b [22]. Several spices and herbs were recently rejected from different countries due to their contamination with *Salmonella* such as laurel leaves and curry powder from Turkey and ginger powder from India [23]. In Lebanon, multiple studies have examined the microbiological quality of spices and herbs available on the market [24,25,26], whereby samples collected, such as whole black pepper, black pepper powder, paprika, curry, and turmeric, exhibited elevated levels of total aerobic mesophilic bacteria, sulfite-reducing anaerobic bacteria, *C. perfringens*, coliforms, *E. coli*, yeasts, and molds. Among spices, cumin seed, scientifically known as *Cuminum cyminum*, is widely recognized and used due to its unique flavor, scent, and beneficial effects for health and healing [27,28,29,30,31,32,33,34,35]. *Escherichia coli*, *Salmonella*, *Clostridium perfringens*, and *Bacillus cereus* can easily contaminate cumin seeds throughout growth, harvest, and manufacture. This contamination can potentially pose a concern to public health [36]. In this context, cumin seeds were selected as a model food to assess the decontamination technique used in this study. Various techniques have been employed to eliminate contaminants from spices and herbs. Traditional techniques like fumigation using ethylene oxide (EtO) have been employed, showing high efficiency in reducing microbial load. However, fumigation has significant drawbacks including the production of cancer-causing toxic substances. Consequently, the European Union prohibited this approach in 1991 [37]. In contrast, according to the American Spice Trade Association (ASTA), EtO is still used to reduce microbial contamination of spices (ASTA, 2024) [38]. Another method, known as gamma irradiation, has been developed, and according to a study conducted by Schotroff et al. on allspice berries, caraway seeds, oregano, and rosemary leaves [39], gamma irradiation reduced *Enterococcus faecium* counts below the detection limit. However, this technology is characterized by low consumer acceptance and legal restrictions in a variety of countries [40] due to the formation of carcinogenic radiolytic compounds such as 2-alkylcyclobutanones (2-ACBs) [41]. Other treatments were followed as well, such as thermal treatment of spices and herbs, although this affected negatively the quality parameters of the product, mostly in the content of polyphenols, volatile components, and color [42]. In order to address these drawbacks, various advanced technologies have been developed, notably ozonation [43], cold plasma [44,45,46], infrared [47], ultraviolet [48], and microwave technologies [49]. Hurdle technologies were applied as well for the decontamination of spices. For instance, El Darra et al. [50] developed a novel model consisting of ozone, ultraviolet, and infrared light and demonstrated its effectivness to decontaminate onion flakes and black pepper inoculated with *Escherichia coli*. These studies have demonstrated promising outcomes in terms of decontaminating spices and herbs but had certain limits, particularly when it came to their use in food products. Therefore, it is crucial to explore alternative decontamination methods for herbs and spices to enhance quality control and ensure their safety for consumption. This will also help improve market access while considering environmental concerns, the connection between microbial standards and product quality, energy usage, and the preservation of product quality by extending its shelf-life.

Recently, a new process called “Intensification of Vaporization by Decompression to the Vacuum” (IVDV) was patented, designed, and developed in the laboratory of the Faculty of Sciences at Saint Joseph University of Beyrouth and Faculty of Arts and Sciences at University of Balamand. This technique was developed in order to enhance the texture of some products such as roasted chickpeas [51] and to improve polyphenol extraction from olive leaves [52].

This study focused on investigating the IVDV procedure as a method for treating inoculated *C. cyminum* seeds. The procedure was achieved by the utilization of various levels of saturated steam pressure (P) and processing time (t). The study aimed to assess the effect of IVDV treatment on the microbial load of spices, color, and oil profile and to evaluate the alterations that took place following IVDV processing. The optimization process based on the response surface methodology (RSM) was used to determine the optimal conditions of treatment by IVDV, resulting in a product that is both safe for consumption and exhibits satisfactory organoleptic properties.

## 2. Materials and Methods

### 2.1. Raw Materials

The *C. cyminum* seeds were purchased from local Lebanese merchants who import seeds from Syria. These seeds were assessed for their microbiological content at the Lebanese Agricultural Research Institute (LARI), which is accredited under the ISO/IEC 17025 standard (ISO/IEC 17025:2017) [53]. The solvents and chemicals used in the experiment were of analytical quality and were supplied by Merck (Darmstadt, Germany) and Sigma-Aldrich (Saint Louis, MO, USA).

### 2.2. Microbial Inoculation

The microbiology laboratory of the Lebanese Agricultural Research Institute, Fanar, Lebanon, provided *Escherichia coli* (ATCC 25922) and *Salmonella* Typhimurium (ATCC 14028) for the purpose of inoculating the *C. cyminum* seeds. The artificial inoculation technique was implemented in order to guarantee an accurate measurement of the contamination level and to ensure we were working with clearly identified strains to test the efficiency of the decontamination process. Stock cultures of microorganisms were stored in Luria broth (Scharlau, Barcelona, Spain) supplemented with 60% glycerol at −18 °C. Each bacterial strain was transferred into tryptic soy broth (Scharlau, Barcelona, Spain) and incubated at 37 °C for 24 ± 2 h. The initial microbial load of the spraying suspension was defined by adjusting its turbidity to McFarland standard #4. Basically, the estimated cell density for each bacterial strain was approximately 1.2 × 10^9^ cfu/mL.

One hundred grams of *C. cyminum* seeds was placed in a sterile polypropylene bag, sprayed with 1 mL of *Salmonella* Typhimurium and 1 mL of *E. coli*, and hand-massaged for 10 min inside a biosafety cabinet to ensure the maximum adherence of the inoculum. The theoretical microbial concentration would then be 1.2 × 10^7^ cfu of each bacterial strain per gram.

### 2.3. IVDV Microbial Inactivation

**Figure 1:**
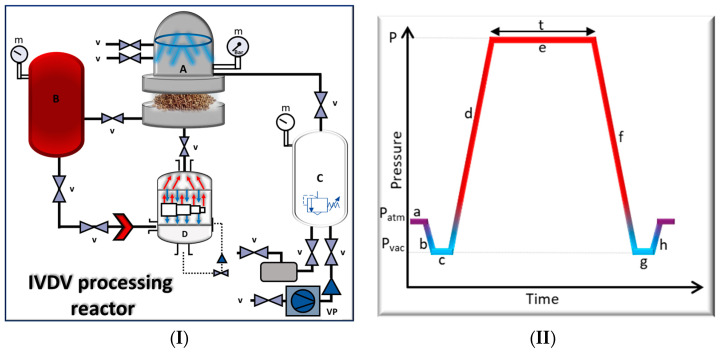
(**I**) Schematic diagram of the IVDV system. A: treatment chamber; B: rapid steam generation system; C: vacuum tank; D: ultra-speed pressure increase system; VP: vacuum pump; v: valve; m: manometer. (**II**) Time–pressure pattern of an IVDV processing cycle. (a) Atmospheric pressure in the treatment chamber, (b) establishment of initial vacuum in the treatment chamber, (c) stay under vacuum, (d) injection of saturated steam, (e) constant pressure treatment, (f) decompression to vacuum, (g) vacuum—air injection—cooling, (h) establishment of atmospheric pressure.

The inoculated samples were treated using the IVDV reactor shown in Figure 1I. It consists of four major parts: treatment chamber (A), steam generator (B), vacuum tank (C), and an ultra-speed pressure increase system (D). The time–pressure pattern of an IVDV processing cycle is illustrated in Figure 1II, where (a) represents the atmospheric pressure in the treatment chamber; (b) establishment of initial vacuum; (c) stay under vacuum; (d) injection of saturated steam (15 bars/s); (e) constant pressure treatment for each run with specific steam pressure and processing time; (f) decompression to vacuum (150 bars/s); (g) vacuum—air injection—cooling, and (h) establishment of atmospheric pressure before retrieving the sample. IVDV is a thermo-mechanical treatment that was originally employed as a method for the texturizing of fruits and vegetables like chickpeas and mango, as well as to improve the efficiency of extracting bioactive molecules from plants [54,55]. In this study, IVDV was used as a novel technique to decontaminate artificially inoculated cumin seeds. A total of 100 g of *C. cyminum* seeds was introduced into the treatment chamber and underwent thermal treatment under a saturated steam pressure that rapidly reached a high pressure with an increasing speed upwards of 15 bars/second. Afterwards, decompression takes place, accompanied by cooling, to prevent the deterioration of the product. The IVDV method is an innovative technology that utilizes rapid compression to effectively and safely treat heat-sensitive goods that have a low tolerance for high temperature lasting only a few seconds.

### 2.4. Bacterial Enumeration

After undergoing IVDV treatment, the treated samples were transferred to sampling bags for enumeration. To quantify the remaining bacterial cells following treatment, the initial sample in the bag (5 g) was diluted ten-fold by adding 45 mL of BPW solution and subsequently homogenized for 1 min in a stomacher (Neutec Group Inc., New York, NY, USA). Blended samples were decimally diluted in 9 mL of BPW; all samples were plated in duplicate onto *Salmonella Shigella* Agar (SS Agar) (Scharlau, Barcelona, Spain) and incubated for 24 h at 37 °C and onto Tryptone Bile Glucuronic Agar TBX (Scharlau, Barcelona, Spain) and incubated at 44 °C for 24 h (ISO 16649-2:2001) for *E. coli* [56].

### 2.5. Extraction of Oils and Their Analyses

#### 2.5.1. Extraction of Essential Oils

The process of extracting essential oils involved the use of 200 g of cumin seeds in a Clevenger-type device for a duration of three hours [4]. The oil was dehydrated using anhydrous sodium sulfate and stored in dark glass vials at a temperature of 4 °C before conducting GC-MS experiments.

#### 2.5.2. Gas Chromatography–Mass Spectrometry (GC–MS) Analysis

The analysis of the essential oil components was conducted using a GC-MS/GC 6890 by N Agilent Technologies. The analysis involved a helium constant flow rate of 1 mL/min, an inlet temperature of 250 °C, an injection volume of 1 µL, and an oven temperature program consisting of three steps: 60 °C increasing at 3 °C/min to 200 °C. The after-run temperature was set at 280 °C for 10 min. The mass spectrometer (Agilent 5975B) was configured with a mass range of 40 to 450 atomic mass units (amu). The ionization energy was set to 70 electron volts (eV). The ion source temperature was set to 230 °C, while the quadrupole temperature was set to 150 °C. A solvent delay of 4 min was implemented, and the transfer line temperature was set to 280 °C. The identification of chemicals was facilitated by employing software (NIST 2.0d; National Institute of Standards and Technology Standard Reference Data Program supplied by Agilent Technologies Germany, Waldbronn, Germany). The constituents were identified by determining their retention time (RT) using a series of n-alkanes (C4–C30) under the same experimental conditions. This was carried out by co-injecting them with either a standard from Sigma-Aldrich or known essential oil constituents. Additionally, a search was conducted using the MS library NIST 05 and the results were compared with the existing MS literature data.

### 2.6. Color Determination

The color of the *C. cyminum* seed samples was assessed before and after IVDV treatment using a colorimeter IRIS (SA Alpha MOS, 31500 Toulouse, France). A 3 g sample was placed in the measuring chamber. The instrument was configured to measure using the CIE system, which is an abbreviation of the International Commission on Illumination (CIE). The measurements were taken for the values of lightness (L*), red/green (a*), and yellow/blue (b*). The total color change (Δ*E*) was computed for each sample by completing ten replications and using the calculation provided below [57]:

(1) $$\Delta E=\sqrt{{\left(L^{*}-{L_{0}}^{*}\right)}^{2}+{\left(a^{*}-{a_{0}}^{*}\right)}^{2}+{\left(b^{*}-{b_{0}}^{*}\right)}^{2}}$$

### 2.7. Experimental Design

This study utilized response surface methodology (RSM) to optimize the IVDV treatment. A rotatable central composite design was created to evaluate the influence of two independent variables, pressure (P) and time (t), on the following response parameters: log reduction for *E. coli* (logN_0_/N*^E. coli^*), log reduction for *Salmonella* Typhimurium (logN_0_/N*^Salmonella^*), and color (L*, a*, b*, and Δ*E*). The fundamental principle of RSM entails methodically completing a sequence of planned experiments in which the values of independent variables are deliberately manipulated within predetermined limits. A dataset is created by measuring the response variable for each combination of input variable values. The experimental outcomes are used to create empirical models that depict the link between the input factors and the response variable. The core of RSM involves generating a response surface, which can be presented as either a graphical depiction or a mathematical equation that demonstrates the connection between the independent factors and the response variable [58,59,60]. A central composite rotatable design consisting of twelve runs including four repeats at the central points was developed to assess the impact of pressure and time on microbial load and Δ*E* as response variables. The pressure varied between 1.48 bar and 3.5 bar, which correspond to 111 and 139 °C, respectively, while the treatment time ranged from 2.5 s to 133.45 s. The values with the greatest and smallest magnitudes were designated as −α and +α, correspondingly (Table 1). The tests’ design and findings were evaluated using STATGRAPHICS Centurion XVII-X64 (Sigma Plus, Virginia Beach, VA, USA).

### 2.8. Statistical Analysis

The bacterial counts underwent a logarithmic transformation before being subjected to statistical analysis. log reductions were determined by subtracting the logarithm of the colony-forming units per gram (cfu/g) in the treated sample from the logarithm of the cfu/g in the untreated control sample. An RSM was used to fit a model for the logarithmic reduction in *E. coli* and *Salmonella* Typhimurium inactivation by IVDV. The colorimeter assay was conducted 10 times to confirm the consistency of the data, while the reproducibility was addressed by performing four repeats at the central level, as recommended by the experimental design. Calculations were performed to determine the mean values and standard deviations. The data underwent statistical analysis using the analysis of variance (ANOVA). A confidence level of 95% was assigned to *p*-values less than 0.05, indicating statistical significance. The treatment procedure was optimized using STATGRAPHICS^®^ Centurion XV software (Statgraphics 18, The Plains, VA, USA).

## 3. Results and Discussion

### 3.1. Response Surface Design

The objective of this study was to identify the optimal values of P and t that would result in the highest log reduction for *E. coli* and *Salmonella* Typhimurium while limiting the color change (Δ*E*) in treated *C. cyminum* seeds. Table 2 displays the experimental design with the operating parameters, and the results of the response parameters, including log reduction for *E. coli* (cfu/g), log reduction for *Salmonella* Typhimurium, and color analysis (L*, a*, b*, and Δ*E*). The initial microbial load of the untreated samples, following the artificial inoculation, was 3 × 10^8^ cfu/g of *E. coli* (ATCC 25922) and 1.08 × 10^7^ cfu/g of *Salmonella* Typhimurium (ATCC 14028). The log reduction values for *E. coli* (logN_0_/N*^E. coli^*) ranged from 0.49 to 3.40 and for *Salmonella* Typhimurium (logN_0_/N*^Salmonella^*) ranged from 0.10 to 3.42. The maximum value of log reduction for both bacterial strains was achieved for run 8, with a pressure of 2.32 bar and a time of 133.45 s. After treatment, the values of L*, a*, and b* exhibited mild variations, where lightness decreased as indicated by a decrease in L* value from 92.00 (untreated sample) to 80.16 (run 6). As for a* and b* values, they increased, indicating a less green and more yellow product, respectively, with values ranging, for a*, from 6.77 in untreated cumin seeds to 10.68 in run 6, and for b*, from 43.01 in untreated cumin seeds to 51.93 in run 8, noting that these changes in color are very mild. Therefore, the target of using RSM for optimization for color analysis was to minimize the Δ*E* and identify the ideal values of P and t that would result in the lowest color change for treated *C. cyminum* seeds.

### 3.2. Experimental Modeling

Table 3 represents the first-order and second-order polynomial equations for *C. cyminum*-treated seeds relating response variables to operating parameters, which were determined through the use of a multiple regression analysis on the experimental data. All response values were found to be consistent with the first- and second-order polynomial models mentioned in Table 3.

### 3.3. Effect of Steam Pressure and Processing Time on Microbiological Load

**Figure 2:**
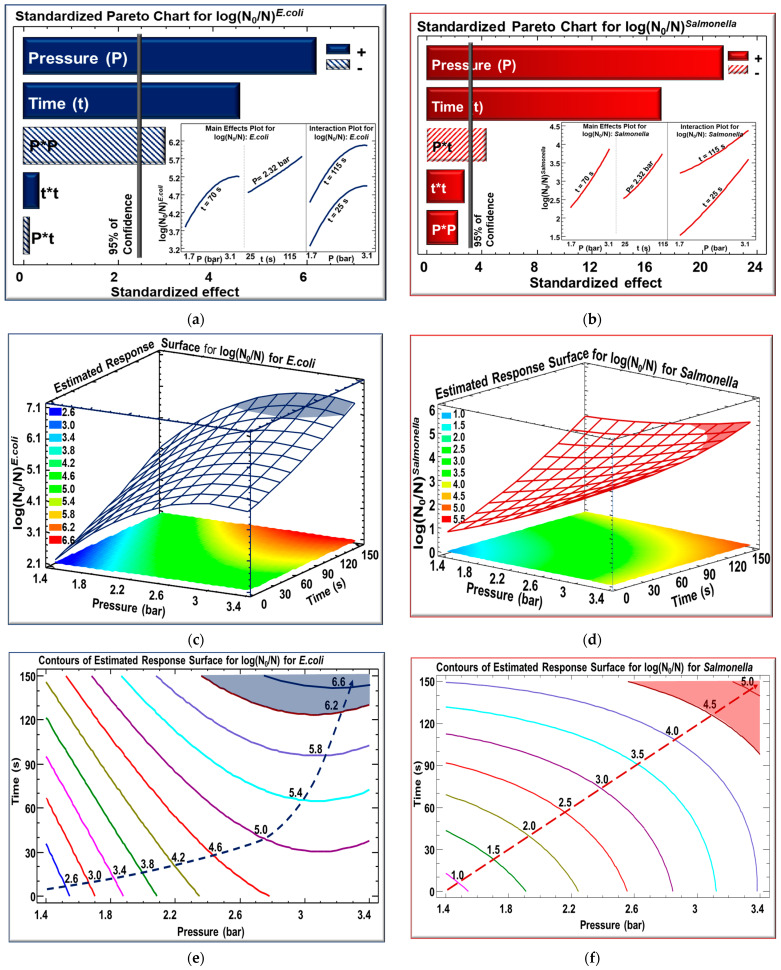
Standardized Pareto charts (**a**,**b**), their corresponding estimated response surface plots (**c**,**d**), and their corresponding estimated response contours (**e**,**f**) of *E. coli* and *Salmonella* Typhimurium for IVDV-treated *C. cyminum* seeds.

The influence of processing parameters, steam pressure (P) and treatment time (t), on the reduction in microorganisms in contaminated cumin seeds was measured and represented using Pareto charts and response surfaces for various response parameters (Figure 2). Figure 2a,b display the Pareto charts illustrating the effect of treatment parameters for *E. coli* (Figure 2a) and *Salmonella* Typhimurium (Figure 2b) reduction. The significance of parameter effects is demonstrated by bars that intersect the vertical line at a confidence level of 95%. The hatched bars indicate a negative effect of the parameters, whilst the remaining bars show a positive effect. In Figure 2a, the linear effect of pressure and time were the most significant and had a positive effect on microbial log reduction for *E. coli*, although pressure had a negative quadratic effect. It is noticeable that pressure of saturated steam was the most significant factor followed by time. Whenever the saturated steam pressure increased, temperature increased as well, reaching a point where stabilization occured. This is clearly identified in inserts showing that when the pressure increased from 1.7 to 3.1 bar and time is fixed at 70 s, the log reduction for *E. coli* increased from 3.7 to 5.2 and then it stabilizes. As for time, when it increased from 25 s to 115 s while setting the pressure at a central point of 2.32 bar, a linear increase was seen without stabilization, which means that more log reduction will be observed when time is increased. The shape of evolution of the log reduction in *E. coli* as a function of pressure at 25 s and at 115 s was similar, which indicated that no interaction exists between P and t. In Figure 2b, Pareto charts revealed the significance of pressure and time having a positive effect on the microbial log reduction in *Salmonella* with a small interaction between parameters. In Figure 2b inserts, a linear increase in log reduction for *Salmonella* from 2.4 to 4 was observed when time was fixed at 70 s and when pressure increased from 1.7 to 3.1 bar. Similarly, when pressure was set at 2.32 bar, a linear increase was observed for log reduction from 2.5 till 3.9 when the time increased from 25 s to 115 s. The shape of evolution for the log reduction in *Salmonella* as a function of pressure when time is fixed at 25 s showed a linear increase, indicating the major effect of temperature on log reduction. However, this effect tends to stabilize when time reached 115 s, which indicates a significant interaction between these two parameters.

Figure 2c,d represent the estimated response surface showing the evolution of log reduction for *E. coli* (Figure 2c) and *Salmonella* (Figure 2d) as a function of pressure and time. The blue-colored region and the red-colored region on the grid represent the optimal zone for log reduction in *E. coli* and for *Salmonella*, respectively. The results are further clearly demonstrated by contour plots depicting the estimated response surface for the microbial reduction in *E. coli* and *Salmonella* Typhimurium as a function of time and pressure, as shown in Figure 2e,f, respectively. Dashed blue lines in Figure 2e and dashed red lines in Figure 2f show how the evolution of log occurred. For instance, in Figure 2f, if we choose the cyan-colored line, at any point on this line, the same response will be obtained with a 3.5 log reduction for *Salmonella* at a different time and pressure, which provides us with the possibility of choosing the couple (P/t), ensuring at the same time a log reduction of 3.5. The findings are consistent with the study conducted by Newkirk et al. [61], who highlighted the effect of time on microbial reduction by comparing the average log reduction in cfu/g of *Salmonella* on inoculated whole black peppercorns, which were pasteurized at 88 ± 5 °C with 97.9 kPa for different periods of times. They concluded that the log reduction in *Salmonella* after using lab-scale vacuum steam pasteurization was significantly affected by the time of treatment (3 min) to assure a 5 log reduction in *Salmonella* in inoculated whole black peppercorns.

### 3.4. Effect of Steam Pressure and Processing Time on Color

**Figure 3:**
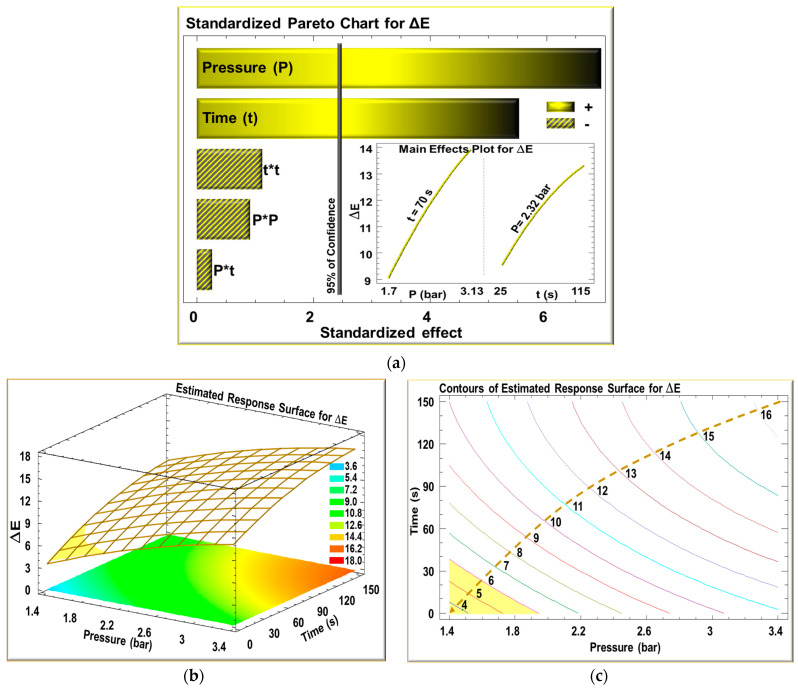
Standardized Pareto chart for Δ*E* (**a**); estimated response surface plot (**b**); estimated response contours (**c**) for color (Δ*E*) of IVDV-treated *C. cyminum* seeds.

**Figure 4:**
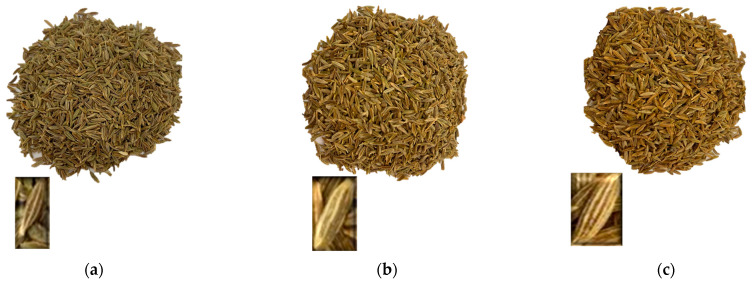
Photos of untreated (**a**) and IVDV-treated (**b**,**c**) *C. cyminum* seeds.

Figure 3 represents the standardized Pareto chart for Δ*E* with the main effect plot for Δ*E*. Color analyses of treated *C. cyminum* for Δ*E* showed that IVDV steam pressure (P) and processing time (t) had a significant positive linear effect on Δ*E*, as demonstrated in the Pareto chat (Figure 3a), noting that whenever the saturated steam pressure increased, the temperature increased as well, which will affect the color. Therefore, IVDV treatment parameters developed a product with different aspects for cumin seeds that is darker, more yellow, and less green (Table 2). This is identified as well in the inserts; when the pressure increased from 1.7 bar to 3.13 bar and time was fixed at 70 s, a linear increase for Δ*E* from 9 to 14 was observed. Similarly, when time increased from 25 s to 115 s, and pressure was fixed at 2.32 bar, Δ*E* increased in the same manner. Moreover, the estimated response surface highlighted the evolution of Δ*E* with a tendency to stabilize. The optimal area is colored in yellow on the grid, where any time and pressure in this designated region will lead to a value for Δ*E* equivalent to 4.1 for treated samples (Figure 3b). This is demonstrated as well in Figure 3c, showing the estimated response contour regions for color relevant for the optimum value. In this figure, the arrow is directed in the opposite way, indicating that whenever Δ*E* is less requested, the pressure and time of the treatment should be decreased. These optimum parameters led to a slight change in L*, a*, and b* values, as previously demonstrated in Table 2 and Figure 4, in which photos of an untreated sample (Figure 4a), an IVDV-treated sample that falls within the optimal area for Δ*E* relative to run 1 (Figure 4b), and an IVDV sample with mild color change for run 6 (Figure 4c) are illustrated. The three photos were taken from the same distance suing a 15″ portable folding light box. A remarkable change in size was observed among the three samples, especially in Figure 4c, which explains the main effect of IVDV in terms of product expansion. This effect was observed as well by Labaky et al. [55], where IVDV treatment increased the expansion ratio of mango and improved its texture and color. Moreover, the slight decrease in color is mainly due to the distribution of pigments on the larger surface of treated products. Thus, this expansion will lead to a higher release of compounds from inside to outside the product during infusion. In accordance, a study conducted by Mrad et al. [51] demonstrated that roasted chickpeas treated with IVDV underwent color change as well and were accepted by panelists through sensory analysis, resulting in a product with desirable organoleptic properties.

### 3.5. Optimization of the Treatment

**Figure 5:**
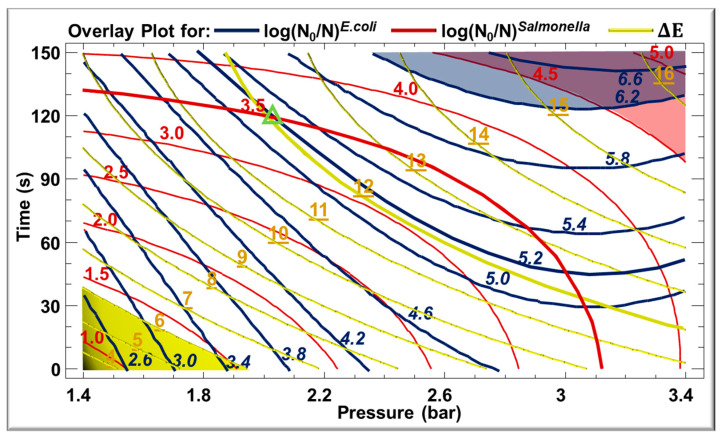
Contours of the estimated response surface of logN_0_/N for *E. coli* and *Salmonella* and for Δ*E* as a function of time and pressure for *C. cyminum* seeds.

Using response surface methodology (RSM), the optimization of each response parameter was realized separately, and the variables standing for the optima are revealed in Table 4. The optimal conditions found to maximize microbial log reduction for *Salmonella* Typhimurium were 3.5 bar and 133.45 s. Similar conditions were obtained for *E. coli* log reduction, with optimum response values of 3.3 bar and 133.5 s. On the other hand, the optimum conditions to minimize Δ*E* were 1.48 bar and 2.5 s since these parameters minimally affected the color of treated *C. cyminum* seeds. The overlay contour plots of the estimated response surface for log reduction for E. coli, log reduction for *Salmonella* Typhimurium, and Δ*E* are shown in Figure 5. The evolution of log *E. coli*, log *Salmonella,* and Δ*E* is represented by blue lines, red lines, and yellow lines, respectively. Moreover, values written in red are for *Salmonella*, in italic blue are for *E. coli*, and in underlined yellow are for Δ*E*. The optimal region for *E. coli* and *Salmonella* are inter-related; therefore, the region intersecting the red and blue colors provides the maximum log reduction for *E. coli* and *Salmonella*, whereas the region colored in yellow represents the minimal change in Δ*E*. Since food safety is of prime importance, no single optimum can be obtained to have a maximum log reduction without affecting the color. Therefore, results shown in this figure can be used as a reference for other researchers seeking to reduce the microbial load with the possibility to choose depending on the microbial load of their product. Accordingly, different parameters can be selected for treatment while deducing the evolution of Δ*E*. For instance, if a product is contaminated with a load of 10^3^ cfu/g of *Salmonella* and the target is to reach 0 cfu/g, referring back to Figure 5, the red line (in bold) where log reduction is 3.5 for *Salmonella* can intersect with the bold blue line with log 5.2 for *E. coli* and the bold yellow line valued 12 for Δ*E* (green triangle). So, a mild change in color will occur, leading to a microbiologically safe product for consumption.

As indicated in Table 4, high R-squared (R^2^) values (91.3, 89.6, and 95.2 for log reduction in *E. coli*, *Salmonella,* and Δ*E*, respectively) for the model indicated an acceptable degree of adequacy between it and the experimental findings.

### 3.6. GC-MS Results

Table 5 provides a comprehensive list of the names of the compounds, their corresponding retention durations, and the peak area for each recognized component identified through arbitrary units. The GC-MS analysis of the essential oils extracted from treated cumin seeds at various IVDV parameters revealed the presence of seven peaks corresponding to monoterpene compounds compared to six peaks for untreated cumin seeds. The main essential oils in the untreated sample were identified with a peak area of 787 for cuminaldehyde, 45.7 for cumenol, 41.5 for γ-terpinene, 37.4 for p-cymene, 17.4 for phellandral, and 10.1 for (+)-4-carene. As for the treated samples, in addition to the six components identified in the untreated sample, d-limonene was present in some samples (samples 1–4) (Supplementary Materials includes the total ion chromatogram of the essential oil of *C. cyminum* seeds for the six IVDV runs). Moreover, the GC-MS analysis revealed that cuminaldehyde was the predominant component in all samples, with the highest quantity seen in run 6, reaching 1260. A study conducted by Ravi et al. [30] on *C. cyminum* identified the chemicals with the greatest concentration as cuminaldehyde (8% to 17%), β-pinene (22% to 27%), p-cymene (23% to 39%), γ-terpinene (11% to 27%), β-myrcene (1.3–1.75%), and p-mentha-1,4-diene-7-ol (1.0–5.5%). Our results are also similar to a study conducted by Rihawy et al. [62] on Syrian *C. cyminum* seeds, where cuminaldehyde was found to be the most prevalent component, accounting for 38.5%. The cumin seeds treated with IVDV, mainly run 5 and 6, showed a higher total content of essential oils compared to the untreated sample. For instance, the content of y-terpinene increased from 41.5 × 10^6^ in the untreated sample to 13.8 × 10^7^ in IVDV run 6. The fundamental reason for this is the rapid decrease in pressure towards a vacuum (~0.003 MPa), which causes the expansion of the sample. This is then followed by a rapid cooling of the sample, which promptly halts its thermal degradation. The expansion effect allows the solvent to penetrate easily in the sample and extract a higher quantity of essential oil. Moreover, the alveolated structure of the expanded matrix accelerates and intensifies transfer of material. The findings align with a prior study conducted by Rezzoug et al. [63] in which the authors showed that employing a technique called Détente Instantanée Contrôlée (DIC), which is a less developed version of IVDV, resulted in an increase in the yield of essential oils compared to the traditional hydrodistillation method.

## 4. Conclusions

*C. cyminum* is a traditional and widely consumed spice known for its distinctive aromatic profile and therapeutic benefits but is also susceptible to contamination during each step of processing. Therefore, in order to provide a microbiologically safe product to consumers without altering the organoleptic properties such as color and without affecting the essential oil profile, a novel technique for decontamination was adopted. In this study, IVDV was used as a treatment technology for artificially inoculated cumin seeds. The aim was to decontaminate cumin seeds while preserving their color and their essential oil profile as much as possible. For this purpose, we studied the effect of IVDV parameters (P, t) on the microbial load, color, and oil profile of artificially inoculated cumin seeds. The optimization of all the response parameters resulted in the following conditions: P = 3.5 bar and t = 133.45 s. The optimized sample resulted in a 99.99% microbial inactivation of *E. coli* and *Salmonella* Typhimurium. It also preserved the oil profile of IVDV-treated samples where all identified monoterpenes were still available after IVDV treatment and slightly affected their color by minimally reducing the lightness and the greenness and increasing the yellowness. This emphasizes the beneficial use of IVDV as a treatment technique for decontamination of herbs and spices without altering the organoleptic properties of the final product.

## Figures and Tables

**Table 1:** Independent variables and their levels used for central composite rotatable design.

Variables	Symbol	Coded Variable Levels				
		−α	−1	0	+1	+α
Steam pressure (bar)	P(T:°C)	1.48 (111)	1.7 (115)	2.32 (125)	3.13 (135)	3.5 (139)
Processing time (s)	t	2.5	25	70	115	133.45

**Table 2:** Central composite arrangement for independent variables and their responses for IVDV-treated seeds.

	IVDV Parameters		Microbial Load		Colorimetric Response Parameters			
Run	P (bar)	t (s)	Log Reduction *E. coli*	Log Reduction *Salmonella* Typhimurium	L*	a*	b*	Δ*E*
Untreated	-	-			92.00	6.77	43.01	
1	1.7	25	0.49	0.10	88.01	7.98	48.56	6.94
2	3.13	25	1.92	2.18	83.08	9.67	50.38	11.93
3	1.7	115	1.13	1.30	85.95	8.91	49.88	9.40
4	3.13	115	2.61	2.60	80.23	10.54	50.14	14.27
5	1.48	70	1.27	1.25	85.89	8.32	48.67	8.47
6	3.5	70	2.57	2.50	80.16	10.68	51.54	15.11
7	2.32	2.5	1.00	0.96	87.10	7.81	48.18	7.20
8	2.32	133.45	3.40	3.42	80.69	10.13	51.93	14.79
9	2.32	70	2.05	1.70	84.19	9.45	51.71	11.99
10	2.32	70	1.48	1.48	86.07	8.43	48.62	8.33
11	2.32	70	1.79	1.70	83.85	9.23	50.47	11.32
12	2.32	70	2.06	2.25	82.50	8.84	48.43	11.13

Central points ranged from 9 to 12.

**Table 3:** Second-order polynomial equations for *C. cyminum*-treated seeds. Log reduction for *E. coli* (logN_0_/N*^E. coli^*), log reduction for *Salmonella* (logN0/N*^Salmonella^*), and color (Δ*E*).

logN_0_/N*^E. Coli^*	=−3.6 + 5.34 × P + 0.012 × t − 0.86 × P^2^
logN_0_/N*^Salmonella^*	=−0.58 + 0.75 × P + 0.023 × t − 0.0069 × P × t
ΔE	=−5.1 + 7.1 × P + 0.08 × t

**Table 4:** Optimum treatment conditions for *C. cyminum* seeds.

Parameters	Optimum Conditions		
	logN_0_/N*^E. coli^*	logN_0_/N*^Salmonella^*	Δ*E*
Pressure (bar)	3.3	3.5	1.48
Time (s)	133.5	133.45	2.5
logN_0_/N*^Salmonella^*	-	5.1	-
logN_0_/N*^E. coli^*	6.35	-	-
Δ*E*	-	-	4.1
R-squared (R^2^)	91.3	89.6	95.2

**Table 5:** The main essential oils extracted from the IVDV-treated cumin seeds.

		Area						
Compounds	RT (min)	Untreated	1	2	3	4	5	6
(+)-4-carene	5.2	10.1	13.8	17.8	17.3	16.3	13.3	25.8
P-cymene	5.4	37.4	33.7	42.4	41.7	36	29.2	62.8
D-limonene	5.5	-	2.6	3.9	3.89	2.97	3.27	-
y-terpinene	6.37	41.5	73.2	114	106	83.1	68.5	138
Cuminaldehyde	13	787	573	656	652	753	882	1260
Phellandral	14	17.4	21.3	24.3	9.4	7.07	7.57	38.8
Cumenol	15	45.7	25.5	32.7	18.5	26.5	33.7	78.9
Total		939	744	881	849	925	1030	1600

## Data Availability

The original contributions presented in the study are included in the article, further inquiries can be directed to the corresponding author.

## Supplementary Materials

The following supporting information can be downloaded at https://www.mdpi.com/article/10.3390/foods13142264/s1, Figure S1: GC-MS chromatogram of untreated *C. cyminum* essential oil, Figure S2: GC-MS chromatogram of treated (run 1) *C. cyminum* essential oil, Figure S3: GC-MS chromatogram of treated (run 2) *C. cyminum* essential oil, Figure S4: GC-MS chromatogram of treated (run 3) *C. cyminum* essential oil, Figure S5: GC-MS chromatogram of treated (run 4) *C. cyminum* essential oil, Figure S6: GC-MS chromatogram of treated (run 5) *C. cyminum* essential oil, Figure S7: GC-MS chromatogram of treated (run 6) *C. cyminum* essential oil.
