# Supplementary material for: Microbial Decontamination of Cuminum cyminum Seeds Using “Intensification of Vaporization by Decompression to the Vacuum”: Effect on Color Parameters and Essential Oil Profile
Source: Foods. 2024 Jul 18;13(14):2264. doi: 10.3390/foods13142264 (PMC11275638; doi:10.3390/foods13142264)
Supplement: Supplementary file 1 [file foods-13-02264-s001.zip › foods-3093354-supplementary.pdf]

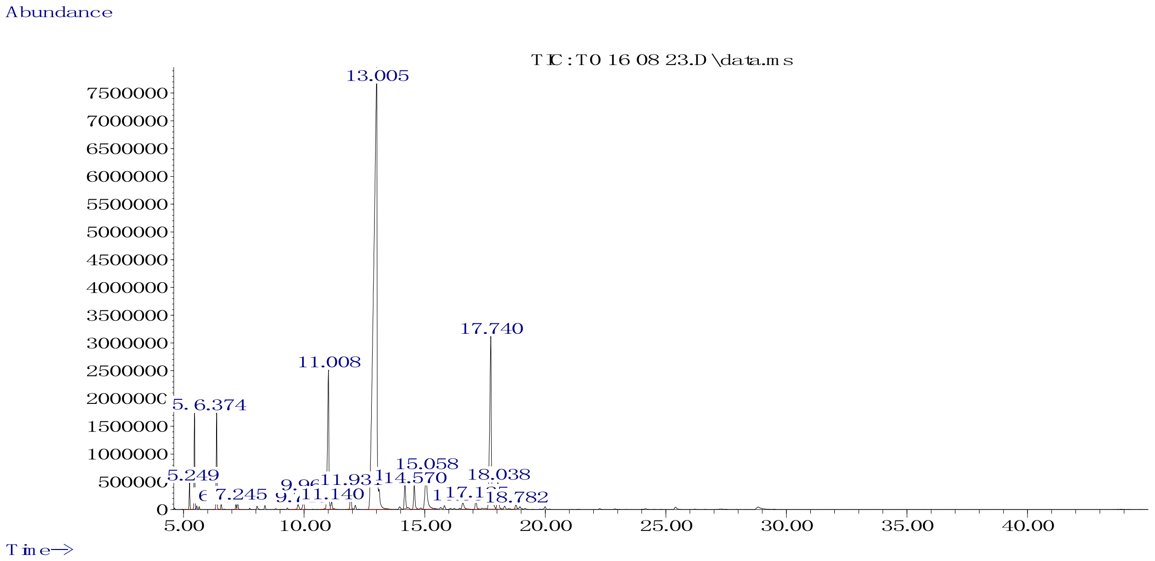

Figure S1: GC-MS chromatogram of untreated *C. cyminum* essential oil

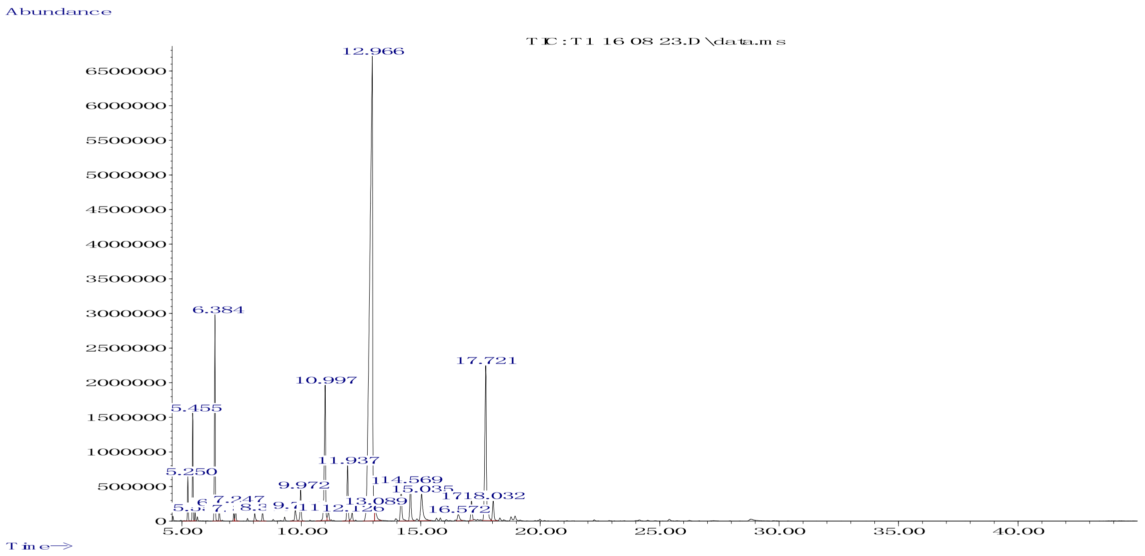

Figure S2: GC-MS chromatogram of treated (run 1) *C. cyminum* essential oil

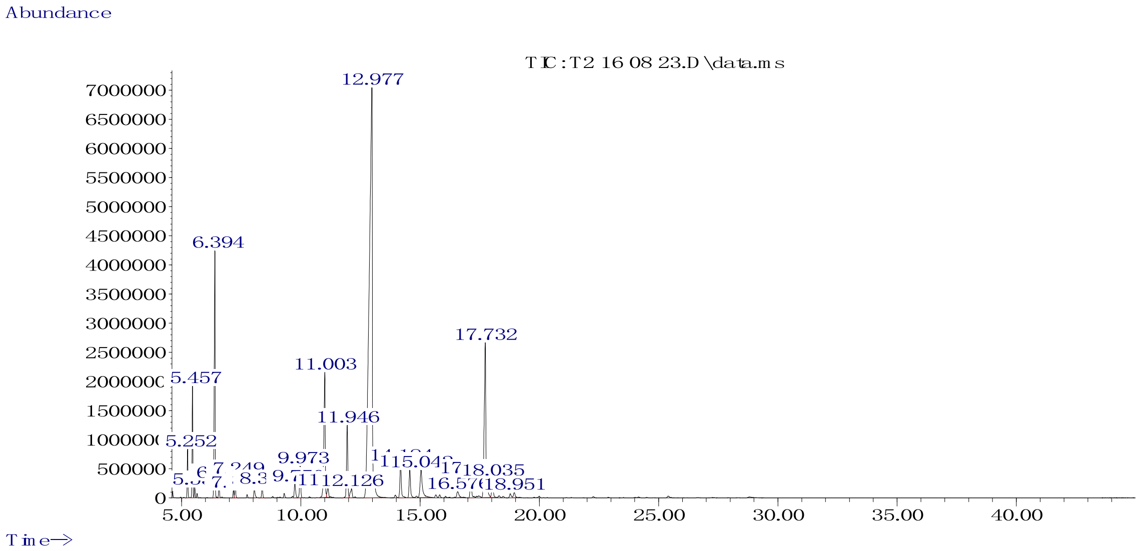

Figure S3: GC-MS chromatogram of treated (run 2) *C. cyminum* essential oil

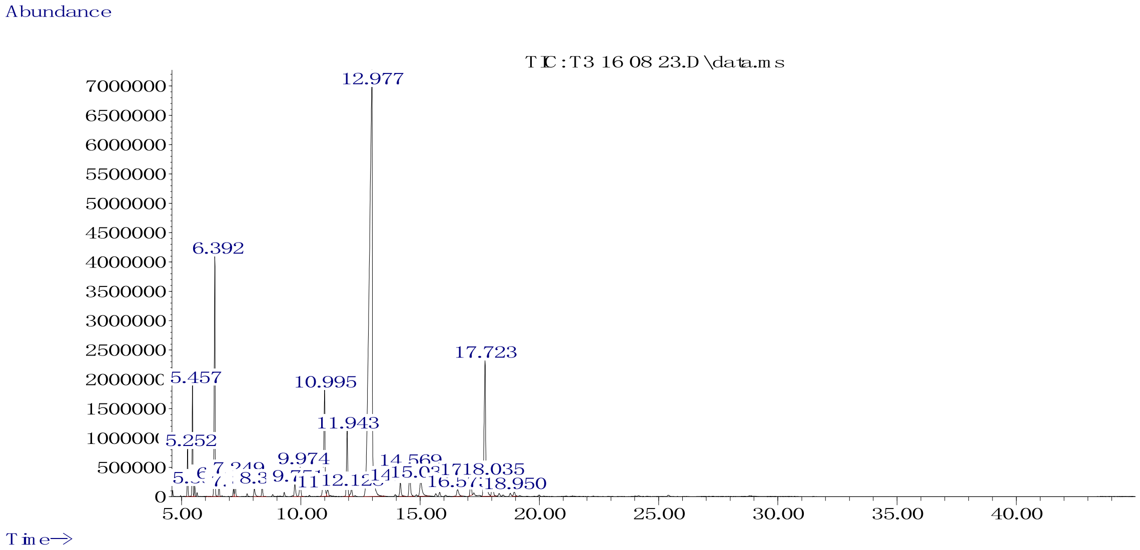

Figure S4: GC-MS chromatogram of treated (run 3) *C. cyminum* essential oil

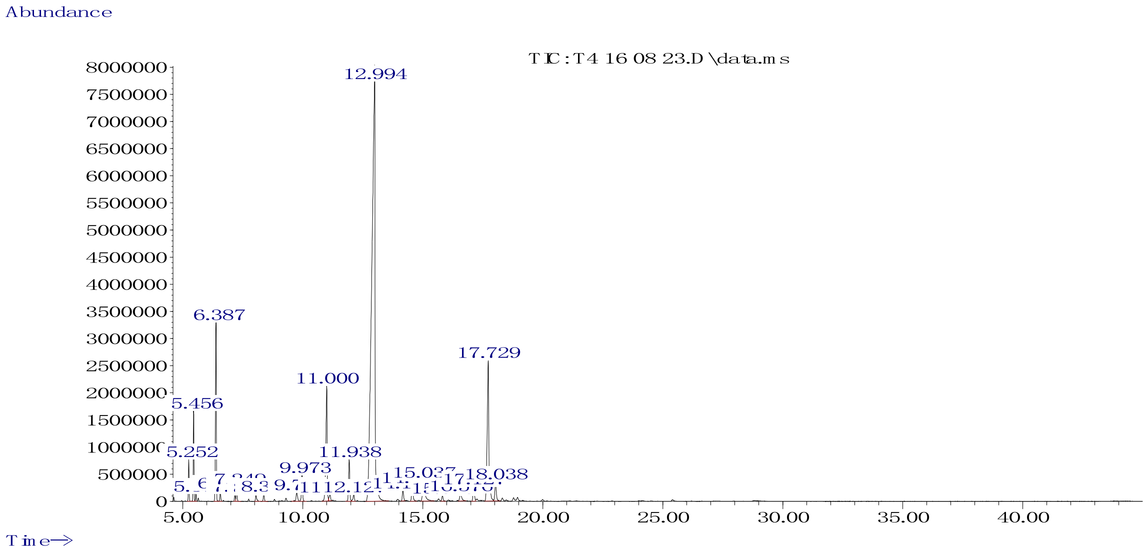

Figure S5: GC-MS chromatogram of treated (run 4) *C. cyminum* essential oil

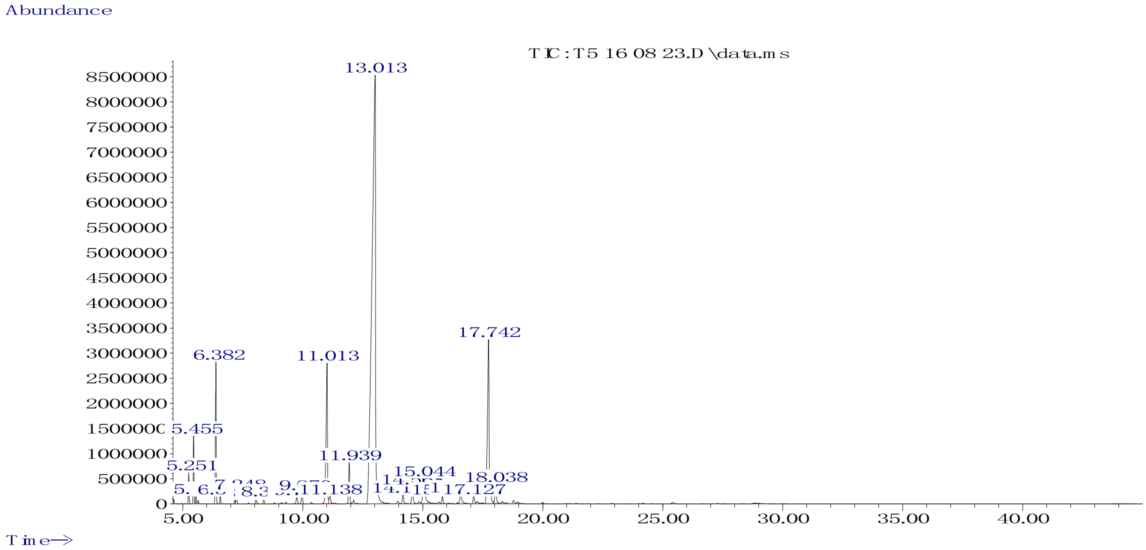

Figure S6: GC-MS chromatogram of treated (run 5) *C. cyminum* essential oil

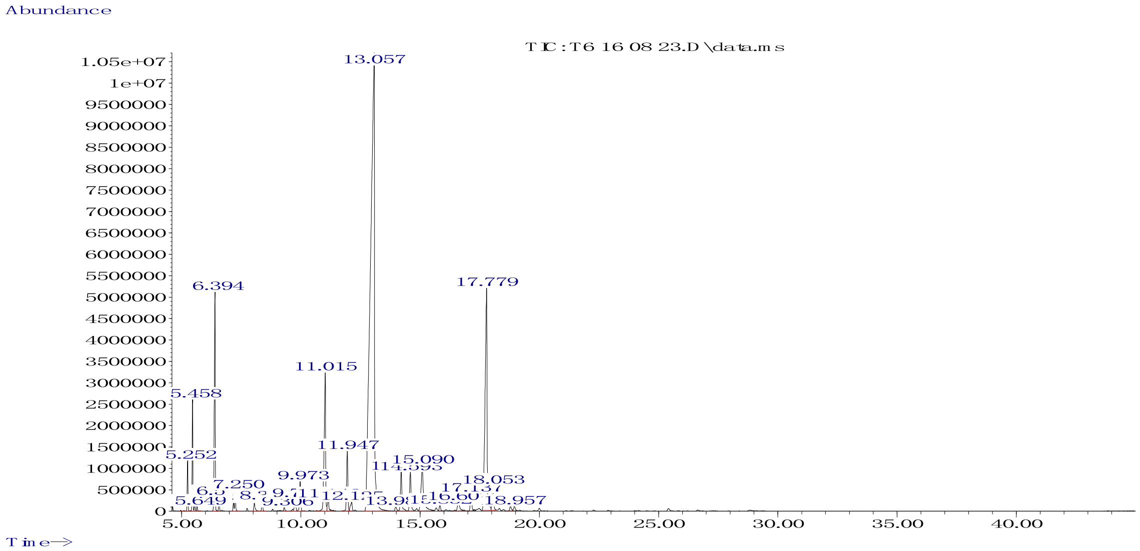

Figure S7: GC-MS chromatogram of treated (run 6) *C. cyminum* essential oil
